# Safety of hookworm infection in individuals with measurable airway responsiveness: a randomized placebo-controlled feasibility study

**DOI:** 10.1111/j.1365-2222.2009.03187.x

**Published:** 2009-07

**Authors:** J Feary, A Venn, A Brown, D Hooi, F H Falcone, K Mortimer, D I Pritchard, J Britton

**Affiliations:** * Division of Epidemiology and Public Health, University of NottinghamUK; † School of Pharmacy, University of NottinghamUK; ‡ Division of Respiratory Medicine, University of NottinghamUK

**Keywords:** airway responsiveness, allergic rhinoconjunctivitis, allergy, hayfever, helminth, hookworm, intervention, *Necator americanus*

## Abstract

**Background:**

Epidemiological evidence suggests that hookworm infection protects against asthma. However, for ethical and safety reasons, before testing this hypothesis in a clinical trial in asthma it is necessary to establish whether experimental hookworm infection might exacerbate airway responsiveness during larval lung migration.

**Objective:**

To determine whether hookworm larval migration through the lungs increases airway responsiveness in allergic individuals with measurable airway responsiveness but not clinical asthma, and investigate the general tolerability of infection and effect on allergic symptoms.

**Methods:**

Thirty individuals with allergic rhinoconjunctivitis and measurable airway responsiveness to adenosine monophosphate (AMP) but not clinically diagnosed asthma were randomized, double-blind to cutaneous administration of either 10 hookworm larvae or histamine placebo, and followed for 12 weeks. The primary outcome was the maximum fall from baseline in provocative dose of inhaled AMP required to reduce 1-s forced expiratory volume by 10% (PD_10_AMP) measured at any time over the 4 weeks after active or placebo infection. Secondary outcomes included peak flow variability in the 4 weeks after infection, rhinoconjunctivitis symptom severity and adverse effect diary scores over the 12-week study period, and change in allergen skin test responses between baseline and 12 weeks.

**Results:**

Mean maximum change in PD_10_AMP from baseline was slightly but not significantly greater in the hookworm than the placebo group (−1.67 and −1.16 doubling doses; mean difference −0.51, 95% confidence interval −1.80 to 0.78, *P*=0.42). Symptom scores of potential adverse effects were more commonly reported in the hookworm group, but infection was generally well tolerated. There were no significant differences in peak-flow variability, rhinoconjunctivitis symptoms or skin test responses between groups.

**Conclusion:**

Hookworm infection did not cause clinically significant exacerbation of airway responsiveness and was well tolerated. Suitably powered trials are now indicated to determine the clinical effectiveness of hookworm infection in allergic rhinoconjunctivitis and asthma.

## Introduction

Almost 800 million people are currently infected with hookworm [1]. Although high levels of hookworm infection can cause significant morbidity and mortality, there is mounting evidence from epidemiological studies that infection is also associated with a reduced risk of asthma [2] and other allergic disorders [3, 4]. This raises the possibility that hookworm infection, particularly at levels of intensity above the threshold of about 50 eggs/g of faeces at which the reduction in risk of wheeze appears to be most marked [5], may also have a therapeutic effect in asthma. If so, experimental hookworm infection may provide an opportunity for the development of new treatment options for this disease [6, 7]. Testing this hypothesis, however, requires first, establishment of the dose of hookworm required to generate the necessary intensity of infection; and second, confirmation that infection is sufficiently safe and well tolerated to make clinical trials feasible. It is also important to determine whether infection induces a natural immune phenotype. A particular safety concern is the possibility of an increase in airway responsiveness during migration of hookworm larvae through the lungs, which typically occurs within 4 weeks of infection [8] and stimulates eotaxin release and a peripheral blood and pulmonary eosinophilia [9], and could therefore cause exacerbation of asthma.

In a pilot study of healthy volunteers, we have established that infection with 10 *Necator americanus* larvae achieved an infection intensity of over 50 eggs/g of faeces, and in the small number of participants involved, was well tolerated [7]. We now report a safety study, performed in the UK and comprising a randomized, placebo-controlled, double-blind trial of hookworm infection in people with allergic rhinoconjunctivitis without clinical symptoms of asthma, but with measureable airway responsiveness to adenosine monophosphate (AMP) below a threshold considered to be indicative of asthma. The study was designed to determine whether hookworm infection exacerbates airway responsiveness at any time during the first 4 weeks after infection. Our secondary objectives were to assess the tolerability of infection and to observe the effect of infection on peak flow variability, rhinoconjunctivitis symptom severity and allergen skin test responses over a 12-week period.

## Materials and methods

### Study participants

Study participants aged 18 years and over with current symptoms of allergic rhinoconjunctivitis were recruited by local advertisement. After excluding those with a diagnosis of asthma or other significant medical disorder, and any women who were pregnant or unwilling to use contraception for the duration of the study, potential participants were invited to attend a screening visit. Recruitment occurred between February and August 2006.

### Screening visit

At the screening visit, the study protocol was explained and written informed consent obtained. Lung function was measured according to international guidelines [10] using a spirometer (Vitalograph, Buckingham, UK) taking the 1-s forced expiratory volume (FEV_1_) as the higher of two values within 100 mL. Subjects were excluded if the FEV_1_ was less than 80% predicted when compared with standard reference ranges [11]. AMP challenge was carried out by inhaling an initial dose of 0.9% saline control, followed by doubling doses (DD) of AMP (Sigma Chemical Co., Poole, Dorset, UK) dissolved in 0.9% saline from 0.115 to 944 μm from a breath-activated dosimeter (MEFAR, Brescia, Italy) set to nebulize for 1 s with a pause of 6 s at a pressure of 152 kPa. FEV_1_ was measured 2 min after each dose. We initially intended to measure airway responsiveness using the provocation dose of AMP required to reduce FEV_1_ by 20% (PD_20_AMP), but found in the course of recruiting for the study that very few volunteers demonstrated this degree of airway responsiveness. We therefore elected to adopt the PD_10_ measure, as this was more easily demonstrable and has been shown to strongly predict PD_20_ [12]. The inhalation challenge was therefore stopped once FEV_1_ had fallen by 10% from the post-saline baseline value, or the maximum dose of AMP had been inhaled [13], and the PD_10_AMP calculated by interpolation between the two last doses on the log dose–response scale [10]. Subjects who did not achieve a fall in FEV_1_ of 10% during the bronchial challenge were excluded from the study.

Venous blood was taken for haemoglobin estimation, differential cell counting, serum albumin and hookworm serology analysis, and participants excluded if they were anaemic or had evidence of past or current hookworm infection. Allergen skin sensitization to *Dermatophagoides pteronyssinus*, cat fur, grass pollen and histamine and saline controls (Diagenics Ltd, Milton Keynes, UK) was measured by standard skin prick test methods [14], with a positive result defined as a mean whal diameter 3 mm greater than the saline control. Subjects without at least one positive result to the three allergen solutions were excluded. Urine analysis for β-HCG (QuickVue, Quidel Corporation, San Diego, CA, USA) was used to confirm that female participants of child-bearing age were not pregnant. An interviewer-administered Juniper Rhinoconjunctivitis Quality of Life Questionnaire (RQLQ) [15] was completed to ensure that subjects had current symptoms and to establish a baseline symptom score.

### Randomization

Subjects fulfilling the entry criteria and who consented to enrolment in the study were seen 1 week later for double-blind randomization to active or placebo infection, allocated in blocks of four according to a computer generated random code. Ten *N. americanus* (L3) larvae in 200 μL of water (active infection) or 200 μL histamine dihydrochloride solution (1.7 mg/mL) (placebo) were administered to an area of skin on the forearm and covered with gauze and a water-tight adhesive dressing, which were kept in place for 24 h. The larvae were obtained by a culture of faecal material from a healthy human source known to be negative for hepatitis B and C and human immunodeficiency virus as described previously [16]. The solutions were administered by an independent member of the research team who was not involved in any of the study measurements, to ensure that the clinical researcher carrying out the protocol measures remained blind to the treatment allocation.

### Follow-up visits

Following randomization, participants were seen by the clinical researcher every week for 4 weeks, then every 2 weeks for a further 8 weeks. To maintain blinding of the clinical researcher, subjects were asked to cover their arm at the site of the dressing application during these visits in case of local skin redness and to discuss any queries relating to the study with a different member of the research team. At each study visit, including the randomization visit, PD_10_AMP was measured as described previously, a Juniper RQLQ completed, and blood taken for haemoglobin, differential cell counts and albumin estimation. To assess the immune phenotype, leucocytes subtypes were determined by flow cytometry, and cytokine and chemokine responses to infection assessed following T cell stimulation. Serological responses to the parasite were assessed by ELISA and western blotting and IgE responses to cat, *D. pteronyssinus* and mixed grass pollens determined to eliminate parasite potentiation of bystander allergic responses; these data will be reported separately.

A stool sample collected within 24 h of the visit was provided, and egg content quantified as described elsewhere [7]. In the week preceding randomization, and for the duration of the study, participants completed a daily diary, recording morning and evening peak expiratory flow rates (PEFR; measured as the best of three attempts) and a symptom score based on a visual analogue scale from 0 (no symptoms) to 10 (maximum possible severity of symptoms) for a range of pre-determined possible adverse effects due to the hookworm. These included skin reactions at the site of infection, tiredness, gastrointestinal and respiratory symptoms. Participants also recorded any use of medication for allergic rhinoconjunctivitis. At the final visit, 12 weeks after randomization, allergen skin tests were repeated. Participants were then unblinded by the researcher who administered the infection or placebo at their randomization visit, and those in the hookworm group given a standard course of mebendazole to eradicate the infection. Stool egg counts and blood eosinophil counts were checked every 2 weeks until egg counts were zero and eosinophils had returned to within normal reference ranges on two successive occasions.

### Medication use

Participants were asked to abstain from the use of antihistamines and steroid nasal sprays for the duration of the study whenever possible. Where occasional usage of antihistamines was unavoidable, participants were provided with a supply of loratidine 10 mg tablets to standardize the antihistamine used, and were instructed to record use in their daily diary. A small proportion of participants requested to take antihistamines on a daily basis due to the severity of their symptoms; and these were asked to substitute their usual medication with loratidine 10 mg taken once daily for the duration of the whole study period; similarly, one participant took a daily steroid nasal spray throughout the entire study.

### Safety and ethics

Data on adverse effects and haemoglobin, eosinophil and albumin levels were monitored as the study progressed by the trial statistician and a study safety committee with *a priori* guidelines on when to withdraw a participant from the study. The study was reviewed and approved by the Nottingham Research Ethics Committee and Research and Development department at the Nottingham University Hospitals NHS Trust.

### Data analysis

The primary outcome variable was the maximum fall in log PD_10_AMP occurring at any time between weeks 1 and 4 after randomization from the value at baseline, expressed in doubling doses. We intended to define the baseline measurement as the mean of the two pre-infection values (screening and week 0); however, because of the learning process that was observed in performing the bronchial challenge, we had concern that the values obtained at the screening visit were not reliable and therefore used the week 0 value as the baseline for the primary analysis. However, the results using the mean of screening and week 0 values as baseline were also computed for comparison. PEFR variability during the first 4 weeks after infection was computed as the two-lowest % mean (mean of the two lowest PEFR values during this period as a percentage of the period mean) [17].

An allergic symptom score was computed at each visit by summing individual symptoms recorded on the Juniper RQLQ (maximum score 168), and summarized over the 12-week study period using area under the curve (AUC) (GraphPad Prism 4, GraphPad Software Inc., San Diego, CA, USA). Change in allergen skin sensitization was computed for each allergen by subtracting the baseline saline-adjusted skin prick result in millimetres from the week 12 result. For each potential adverse effect, the mean daily score was computed for both the whole 12 weeks and also a pre-determined ‘high-risk’ period chosen as the period during which time we predicted the symptoms were most likely to occur. These periods were days 1–21 for localized skin reactions as observed in our previous study [7], days 1–28 for respiratory symptoms coinciding with the period of larval lung migration in the hookworm lifecycle [8], and days 29–70 for gastrointestinal symptoms and tiredness correlating with the period of elevated eosinophil counts which can result in an eosinophilic gastroenteritis [18]. This was to ensure that the magnitude of effect of important adverse symptoms was not diluted by averaging over the full 12 weeks of the study.

All data were analysed using in SPSS version 14.0 (SPSS Inc., Chicago, IL, USA). With the exception of those for adverse effects, all variables were compared between the two groups using the independent samples *t*-test, and multiple linear regression used to adjust for any baseline differences in demographics. The PEFR analysis was repeated excluding any participants who had provided less than 75% of potential readings during the first 4 weeks. The logarithm of the Juniper RQLQ AUC variable was taken to obtain a normal distribution and multiple linear regression carried out to additionally adjust for baseline values. The analysis was repeated excluding participants who used antihistamines more than three times per week (unless used daily). Adverse effects variables were not normally distributed and could not be transformed, so the two groups were compared using the non-parametric Mann–Whitney *U*-test.

Our primary objective was to detect a clinically significant increase in airway responsiveness, which we defined *a priori* to be of one doubling dose or more in magnitude, in the active relative to the placebo group. We estimated that a sample size of 30 (15 in each group) would provide approximately 80% power to detect a difference of this magnitude in the maximum fall in PD_10_AMP between active and placebo groups, assuming a similar repeatability to that reported for PD_20_AMP [13, 19].

## Results

**Fig. 1:**
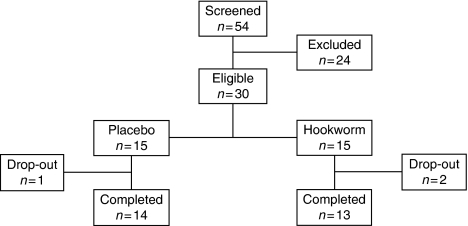
Flow chart of study participants.

Thirty people participated in the study: 15 were randomized to active hookworm infection and 15 to placebo (Fig. 1). There were more current smokers in the placebo group, but otherwise, the demographic characteristics of the two groups were similar (Table 1). Three participants withdrew from the study and were subsequently unblinded: one from the placebo group on day 6 due to an inter-current viral illness; one from the hookworm group on day 12 after becoming pregnant despite a negative pregnancy test at entry into the study and use of contraception; and one from the hookworm group on day 40 due to abdominal pain and diarrhoea, and was treated with mebendazole to eradicate the infection (Fig. 1). The participant who withdrew because of pregnancy kept the infection and completed an otherwise uneventful and successful pregnancy. Exclusion of data from these individuals did not appreciably change the overall demographic characteristics of the two groups. Four participants were unable to attend the week 12 visit and were therefore seen at the earliest possible occasion which was during week 13 for three of them and week 16 (day 112) for one.

**Table 1:** Demographics and baseline characteristics of study participants

	Hookworm (*n*=15)	Placebo (*n*=15)
Males (%)	9 (60)	9 (60)
Mean age (years) at entry (SD)	30.3 (8.97)	33.2 (8.82)
Current smoker (%)	3 (20)	6 (40)
Caucasian (%)	13 (87)	15 (100)
Median PD_10_AMP at screening (IQR)*	13.3 (4.3, 48.3)	24.8 (9.2, 68.8)
Median PD_10_AMP at week 0 (IQR)†	19.8 (6.4, 52.1)	42.1 (23.6, 102.2)

* *n*=13 for each group.

† *n*=14 for hookworm group, *n*=13 for placebo group.

### Airway responsiveness

Data on airway responsiveness over the first for 4 weeks following randomization were available for 28 participants. The mean of the maximum fall in PD_10_AMP during this period was slightly greater in the hookworm than the placebo group (−1.67 and −1.16 DD, respectively), but the difference between the groups was small (−0.51 DD, 95% CI −1.80 to 0.78) and not statistically significant either before or after adjusting for the baseline difference in smoking status (*P*=0.42 and 0.34, respectively, Table 2). The individual maximum changes ranged from −5.55 to 0.81 DD in the hookworm group and −4.26 to 1.47 DD in the placebo group. Peak flow variability over the first 4 weeks was slightly less (i.e. closer to 100%) in the hookworm group compared with placebo, but not significantly so (adjusted mean difference 3.62%, 95% CI −0.66 to 7.90%) (Table 2).

**Table 2:** Respiratory outcomes measured over the first 4 weeks following randomization

	Hookworm mean (SD) (*n*=14)	Placebo mean (SD) (*n*=14)	Mean difference (95% CI)	*P*-value	Adjusted* mean difference (95% CI)	*P*-value
Change in bronchial reactivity using week 0 as baseline (DD PD_10_AMP)	−1.67 (1.72)	−1.16 (1.60)	−0.51 (−1.80, 0.78)	0.42	−0.63 (−1.97, 0.70)	0.34
Change in bronchial reactivity using mean of screening and week 0 values as baseline (DD PD_10_AMP)	−1.52 (1.55)	−0.62 (1.92)	−0.89 (−2.25, 0.46)	0.19	−0.99 (−2.40, 0.43)	0.16
Peak flow variability (Two-lowest % mean)	92.31 (3.73)	89.30 (6.70)	3.01 (−1.21, 7.22)	0.15	3.62 (−0.66, 7.90)	0.09

* Adjusted for smoking status; SD, standard deviation; 95% CI, 95% confidence interval.

### Allergic disease outcomes

The hookworm group reported more symptoms of rhinoconjunctivitis over the 12-week study period than the placebo group, but again this difference was small and not statistically significant either before or after adjustment for smoking and baseline score (Table 3), or after excluding those who had used antihistamines on more than three occasions in a 1 week period (unless taken daily; adjusted mean difference log AUC 0.42, 95% CI −0.49 to1.32). There was no significant difference in change in the magnitude of the wheal response to any of the individual allergens tested (Table 3). The confidence intervals for all these outcomes did not exclude a beneficial effect.

**Table 3:** Allergic outcomes measured over the 12-week study period

	Hookworm mean (SD) (*n*=13)	Placebo mean (SD) (*n*=14)	Mean difference (95% CI)	*P*-value	Adjusted* mean difference (95% CI)	*P*-value
Juniper RQLQ score (log AUC)	6.01 (0.82)	5.68 (0.85)	0.33 (−0.33, 1.00)	0.31	0.26 (−0.45, 0.97)	0.46
Change in grass SPT reaction (mm)	0.73 (2.65)	0.11 (2.65)	0.62 (−1.48, 2.73)	0.55	1.18 (−0.94, 3.30)	0.26
Change in cat fur SPT reaction (mm)	−0.27 (2.09)	−0.75 (1.83)	0.48 (−1.07, 2.03)	0.53	0.64 (−1.01, 2.29)	0.43
Change in DP SPT reaction (mm)	1.27 (2.19)	0.54 (2.59)	0.74 (−1.18, 2.64)	0.44	0.85 (−1.19, 2.90)	0.40

* All adjusted for smoking status; Juniper RQLQ additionally adjusted for baseline score.

95% CI, 95% confidence interval; DP, *Dermatophagoides pteronyssinus*.

### Adverse effects

**Fig. 2:**
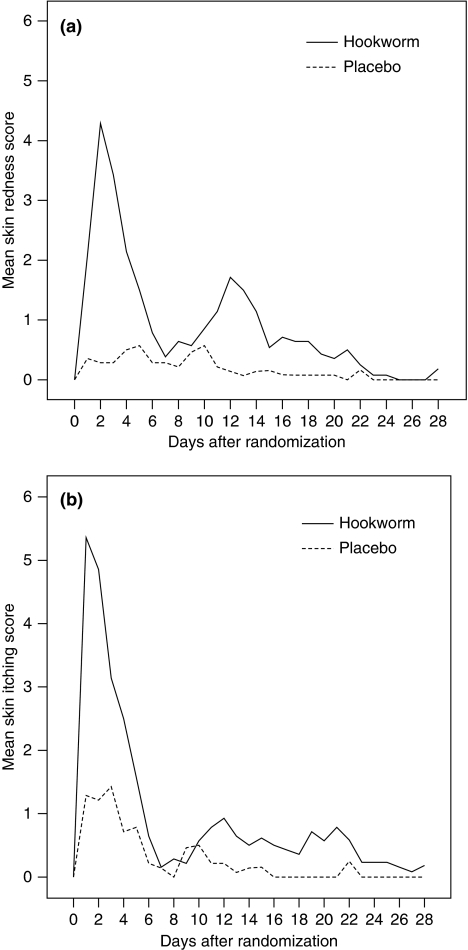
Skin symptoms measured on a visual analogue scale (0–10) over the first 4 weeks after randomization: (a) skin redness, (b) skin itching.

Table 4 shows the reported symptom scores for the potential adverse effects attributable to the hookworm. Both localized skin itching and redness were significantly higher in the hookworm group compared with placebo group, particularly during the high risk period [difference in medians for mean daily score 1.02 (*P*=0.001) and 1.12 (*P*<0.001), respectively; Table 4 and Fig. 2]. These symptoms peaked in the hookworm group in the first week with some subjects experiencing a second, less marked peak, in week 2 as previously described [7]. For the non-skin symptoms, there was more of a range of different participant experiences with many reporting mean daily scores of zero (i.e. no symptoms). Scores tended to be higher in the hookworm than the placebo group but the magnitude of the differences were fairly small. A significant difference was seen for indigestion (difference in medians 0.1, *P*=0.03) during the high-risk period, and a borderline significant effect was seen for abdominal pain (difference in medians 0.48, *P*=0.06). Breathlessness was also higher in the hookworm group but not statistically so, and during the high-risk period this difference was even less marked. Notably, there was no difference between the two groups of the other respiratory symptoms (Table 4).

**Table 4:** Adverse effects reported in subjects with and without hookworm infection

	Mean daily score (scale 0–10) over total 12-week period				Mean daily score (scale 0–10) over high-risk period†			
	Median (range)				Median (range)			
Symptoms	Hookworm group	Placebo group	Difference in medians	*P*-value	Hookworm group	Placebo group	Difference in medians	*P*-value
Localized skin itching	0.30 (0.05–1.10)	0 (0–1.32)	0.30	0.003*	1.12 (0.19–3.11)	0 (0–1.76)	1.12	0.001*
Localized skin redness	0.22 (0.02–1.04)	0 (0–0.48)	0.22	0.001*	1.02 (0.10–5.00)	0 (0–1.90)	1.02	<0.001*
Wheeze	0.30 (0–1.11)	0.07 (0–0.98)	0.23	0.14	0.36 (0–2.07)	0.14 (0–2.00)	0.22	0.29
Cough	0.22 (0–1.02)	0.10 (0–1.54)	0.12	0.56	0.43 (0–1.35)	0.11 (0–2.79)	0.32	0.30
Breathlessness	0.14 (0–6.04)	0 (0–0.98)	0.14	0.07	0.05 (0–5.14)	0 (0–1.86)	0.05	0.34
Nausea	0.17 (0–2.45)	0 (0–1.85)	0.17	0.10	0 (0–4.72)	0 (0–2.07)	0	0.13
Diarrhea	0.12 (0–3.37)	0.11 (0–3.88)	0.01	0.59	0.06 (0–5.64)	0.06 (0–3.95)	0	0.84
Abdominal pain	0.24 (0–3.81)	0.02 (0–3.00)	0.22	0.06	0.48 (0–5.92)	0 (0–3.64)	0.48	0.06
Flatulence	0.28 (0–1.76)	0.13 (0–2.62)	0.15	0.36	0.31 (0–1.98)	0.05 (0–3.05)	0.26	0.16
Indigestion	0.11 (0–2.39)	0 (0–0.87)	0.11	0.02*	0.10 (0–3.92)	0 (0–1.19)	0.10	0.03*
Loss of appetite	0.14 (0–2.25)	0.03 (0–2.21)	0.11	0.67	0.24 (0–4.28)	0 (0–2.60)	0.24	0.30
Tiredness	0.86 (0–6.34)	0.14 (0–2.99)	0.72	0.25	0.41 (0–6.55)	0.15 (0–2.93)	0.26	0.51

* *P*<0.05 (*P*-value for Mann–Whitney *U*-test).

† High-risk periods: localized skin symptoms (days 1–21), respiratory symptoms (days 1–28), gastrointestinal symptoms and tiredness (days 29–70).

Range=minimum–maximum.

All participants had haemoglobin and albumin levels within the normal ranges at entry into the study. No clinically important falls in haemoglobin were seen in either group, with the maximum fall in haemoglobin from baseline being 1.4 g/dL in the hookworm group and 0.8 g/dL in the placebo group. Similarly, there was no suggestion of any significant changes in serum albumin levels with the greatest fall from baseline being 7 g/dL in both groups, to a minimum of 36 g/dL and 31 g/dL in the hookworm and placebo groups, respectively.

### Effect on eosinophil levels

**Fig. 3:**
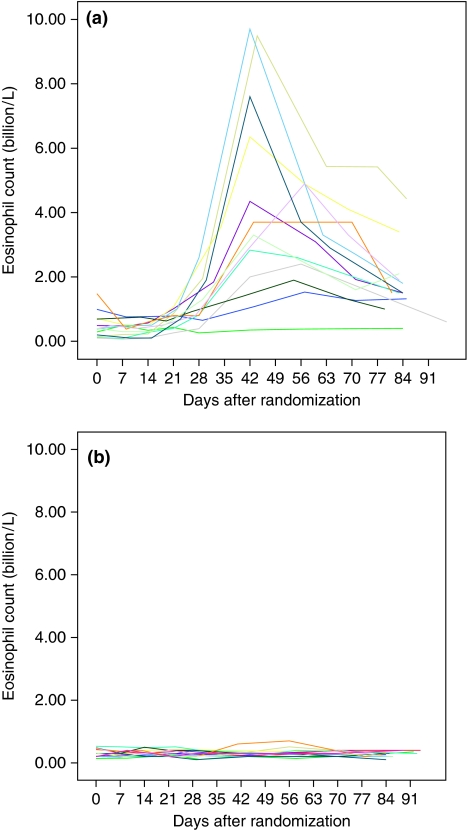
Individuals' peripheral blood eosinophil counts over the 12-week study period: (a) hookworm group, (b) placebo group.

Twelve of the 13 participants in the hookworm group who completed the study had a rise in eosinophil counts, which typically started between 21 and 28 days after infection and reached a peak at weeks 6–8 (days 42–56) (Fig. 3) with maximum counts ranging from 1.53 × 10^9^ to 9.70 × 10^9^ L^−1^. All eosinophil counts decreased after this time but had not returned to baseline values by the end of the study at week 12 (day 84) (Fig. 3). The change in eosinophil counts was reflected by a rise in total white cell counts, which also peaked during this time with a maximum eosinophilia (% eosinophil count/total white cell count) ranging between 21% and 60%; no change was seen in numbers of any other leucocyte type. There was no rise in the eosinophil counts in any of participants in the placebo group.

### Egg counts

Eggs were found in faecal samples of nine participants in the hookworm group, appearing at either week 6 (six people) or 8 (three people) after infection. The presence of these eggs was confirmed by culturing faecal material obtained at week 12 to detect presence of infective larvae. Three of the four participants in the hookworm group with negative stool samples had a rise in their eosinophil count (peaks of 3.3–9.6 × 10^9^ L^−1^), implying that the hookworm larvae had reached the bowel and successfully developed into adult worms. In all nine participants with positive samples, greater than 50 eggs/g of faeces were found on each occasion. Eggs were not seen in any participants in the placebo group. Repeating our primary outcome analysis after excluding the four subjects without eggs in their faeces yielded similar results (mean change in DD PD_10_AMP for hookworm group −0.62: adjusted mean difference −0.81, 95% CI −2.12 to 0.51).

### Assessment of subject blinding

All participants were asked at their final study visit, before being unblinded, if they thought they knew whether they had received hookworm or placebo infection. Of the 14 individuals in the placebo group who completed the study, three correctly thought they had received placebo, five that they had received hookworm, and six did not know. Of the 13 with hookworm infection who completed the study, eight correctly thought that they had received hookworm, two thought they had received placebo, and three did not know.

### Follow-up

All participants who received hookworm were provided with mebendazole at the end of the study to eradicate the infection. However, 11 of the 13 participants who completed the study chose not to take the treatment, citing either a perceived improvement in hayfever symptoms, or that they wished to see if they had a change in symptoms the following year. In accordance with our protocol, participants in the placebo group were offered hookworm infection at the end of the trial, of whom 11 chose to be infected.

## Discussion

To our knowledge, this is the first reported randomized placebo-controlled double-blind study of hookworm infection in people with allergic disease. The study was carried out in preparation for a therapeutic trial in asthma, in accordance with our ethics submission and approval, to look for evidence of any clinically important increase in airway responsiveness during lung migration of larvae. We used the dose we have shown to be well tolerated and which results in an infection intensity of over 50 eggs/g of faeces [7], and which in turn was associated with a reduced risk of wheeze in our observational study [5]. We studied participants with allergic rhinoconjunctivitis and measurable airway responsiveness, but without clinical asthma [20], so that we could look for an increase in airway responsiveness in the active treatment group while minimizing the risk of serious clinical exacerbation of asthma. Participants were also monitored for any symptoms potentially attributable to the hookworm infection. In addition, although designed and powered to detect effects on airway responsiveness, our study also provided an opportunity to explore the effect of infection on rhinoconjunctivitis symptom severity and allergen skin sensitivity.

The procedures we adopted to ensure that participants and clinical investigators remained blind to treatment allocation were successful for the investigator, and predominantly so for participants. Most of those participants who did correctly guess their treatment allocation were in the active treatment group, and usually based their judgment on seeing entry portals on their arm after infection, or the presence of gastrointestinal disturbance. The histamine solution used as a placebo did cause local redness and itching as reflected in the adverse symptoms scores, but this lasted for a shorter period than the local symptoms due to hookworm. We were able to confirm hookworm infection by the presence of eggs in faeces for nine of our infected group, and of the other six participants randomized to hookworm, two withdrew before eggs were expected to be seen in faeces, and three demonstrated increases in eosinophil counts similar to those with proven infection, suggesting that they were actively infected but with same-sex organisms or with non-fecund females. It therefore appears that our infection process failed in only one participant in the hookworm group in whom there was no evidence of active infection. However, excluding this individual or all participants with negative egg counts from the analyses had no material impact on the findings of the study.

The larval pulmonary migration phase of the *N. americanus* lifecycle typically occurs during the first 4 weeks after infection. The stage 3 larvae travel via the blood circulation until they reach the pulmonary vasculature; here, they cross into the alveoli, migrate up the proximal airways, are expectorated and then swallowed. Once in the duodenum, they mature to reach adult status at around 6–8 weeks after infection [21]. While there is some evidence that other intestinal helminths, such as *Ascaris*, may actually exacerbate asthma symptoms [22, 23] and that this may be reversed by parasite eradication therapy [24], asthma exacerbation does not appear to be a problem with hookworm infection [2]. However, studies in animals have demonstrated a pronounced Th2 phenotype associated with this phase of the lifecycle [9, 25], indicating that exacerbation of clinical disease may occur. Furthermore, bronchoscopic investigation before and during larval pulmonary migration in four normal volunteers who had each received 50 infective *N. americanus* larvae demonstrated bronchial mucosal erythema and in one case elevated eosinophils in bronchoalveolar lavage fluid [26]. We therefore felt it essential to carry out an assessment of the effect of hookworm infection on airway responsiveness before proceeding to a clinical trial in asthma.

Because it is not possible to predict when lung migration will take place in any individual subject, we used the largest observed increase in airway responsiveness at any time during the first 4 weeks after infection as our primary outcome. We observed a small increase in airway responsiveness in the hookworm group relative to placebo, estimated to be less than one doubling dose of AMP in magnitude, but this effect was not statistically significant. Furthermore, this size of effect is unlikely to be clinically significant considering the magnitude of normal repeatability for airway responsiveness to bronchial challenge testing is large (95% confidence limits of approximately ±1.5 doubling doses for 2-week repeated challenge to methacholine) [10]. The magnitude of effect we observed is also less than that seen to be associated with established therapies for asthma [27, 28]. During the screening visits, we observed that certain participants took some time to learn how to use the dosimeter properly resulting in unreliable screening measurements of their bronchial responsiveness. We therefore elected to use the week 0 results as the baseline measurement for our primary analysis but also presented the results using the mean of the screening and week 0 values as baseline as per protocol (Table 2). In the hookworm group, the resulting mean value of the primary outcome variable, maximum fall in PD_10_ was similar using the two different definitions for baseline, but less so in the placebo group where the mean value was lower when the definition included the screening data (−0.62 DD) than when based on week 0 data alone (−0.16 DD; Table 2). Further exploration revealed that this discrepancy was primarily due to one individual who responded rapidly to AMP at screening and thus had a very low value, but who had a much higher value at week 0; exclusion of this subject from the analysis resulted in a similar estimated mean difference between hookworm and placebo groups regardless of baseline definition (−0.51 DD using week 0 as baseline and −0.55 using mean of screening and week 0). In addition, neither results for peak-flow variability, nor the reporting of respiratory symptoms showed any evidence to suggest that hookworm infection increased airway responsiveness and thus indicates that infection with 10 larvae is unlikely to exacerbate asthma. The adverse effects reported by participants who were infected with hookworm were principally skin itching and gastrointestinal disturbance, and in the majority of cases were mild with only one person choosing to withdraw from the study as a consequence. Our study thus indicates that this level of infection is likely to be tolerated by the majority of participants in trials, and that exacerbation of asthma is unlikely.

Previous observational studies have suggested that allergic rhinoconjunctivitis is less common in individuals infected with intestinal parasites [29, 30], and an early anecdotal report described an improvement in hayfever symptoms after self-infection [31]. Our study did not detect evidence of a significant improvement in rhinoconjunctivitis symptoms or allergen skin test responses in the infected group relative to the controls, but was not powered or specifically designed to do so; our objective was to identify clinically relevant exacerbation of airway responsiveness. Furthermore, some evidence indicates that helminth infection after allergen exposure may actually lead to a potentiated allergic response and a possible worsening of symptoms as a result, and therefore, in order to suppress allergy would require helminth infection to precede allergen exposure [32, 33]. We did not make allowance for this in the design of our study, but because we have demonstrated that hookworm infection did not potentiate IgE responses to the environmental allergens to which the subjects were sensitized (data not shown). An exploratory analysis of the effects of infection on immune regulation will be reported elsewhere. The clinical effectiveness of hookworm infection in allergic rhinitis therefore needs to be tested in larger studies, in which infection is timed to precede the advent of seasonal allergy, though clinical trials in adults may still show no effect if the protective effect of hookworm infection described in observational studies arises from infection in the early years of life rather than current infection in adults with an already established allergic phenotype. However, the findings of this trial indicate that further definitive trials are feasible and likely to be well tolerated, and that it is also safe to proceed with studies to determine whether infection is effective in the management of asthma.

## References

[1] Bethony J, Brooker S, Albonico M (2006). Soil-transmitted helminth infections: ascariasis, trichuriasis, and hookworm. Lancet.

[2] Leonardi-Bee J, Pritchard D, Britton J (2006). Asthma and current intestinal parasite infection: systematic review and meta-analysis. Am J Respir Crit Care Med.

[3] Cooper PJ, Chico ME, Rodrigues LC (2003). Reduced risk of atopy among school-age children infected with geohelminth parasites in a rural area of the tropics. J Allergy Clin Immunol.

[4] Davey G, Venn A, Belete H, Berhane Y, Britton J (2005). Wheeze, allergic sensitization and geohelminth infection in Butajira, Ethiopia. Clin Exp Allergy.

[5] Scrivener S, Yemaneberhan H, Zebenigus M (2001). Independent effects of intestinal parasite infection and domestic allergen exposure on risk of wheeze in Ethiopia: a nested case–control study. Lancet.

[6] Falcone FH, Pritchard DI (2005). Parasite role reversal: worms on trial. Trends Parasitol.

[7] Mortimer K, Brown A, Feary J (2006). Dose-ranging study for trials of therapeutic infection with *Necator americanus* in humans. Am J Trop Med Hyg.

[8] Hotez PJ, Brooker S, Bethony JM, Bottazzi ME, Loukas A, Xiao S (2004). Hookworm infection. N Engl J Med.

[9] Culley FJ, Brown A, Girod N, Pritchard DI, Williams TJ (2002). Innate and cognate mechanisms of pulmonary eosinophilia in helminth infection. Eur J Immunol.

[10] Crapo RO, Casaburi R, Coates AL (2000). Guidelines for methacholine and exercise challenge testing-1999. This official statement of the American Thoracic Society was adopted by the ATS Board of Directors, July 1999. Am J Respir Crit Care Med.

[11] Quanjer PH (1983). Standardized lung function testing. Report working party. Bull Eur Physiopathol Respir.

[12] Fardon TC, Fardon EJ, Hodge MR, Lipworth BJ (2004). Comparative cutoff points for adenosine monophosphate and methacholine challenge testing. Ann Allergy Asthma Immunol.

[13] Phillips K, Oborne J, Harrison TW, Tattersfield AE (2004). Use of sequential quadrupling dose regimens to study efficacy of inhaled corticosteroids in asthma. Thorax.

[14] Zawodniak AF, Kupczyk MF, Gorski PF, Kuna P (2003). Comparison of standard and modified SPT method. Allergy.

[15] Juniper EF, Guyatt GH (1991). Development and testing of a new measure of health status for clinical trials in rhinoconjunctivitis. Clin Exp Allergy.

[16] Kumar S, Pritchard DI (1992). The partial characterization of proteases present in the excretory/secretory products and exsheathing fluid of the infective (L3) larva of *Necator americanus*. Int J Parasitol.

[17] Siersted HC, Hansen HS, Hansen NC, Hyldebrandt N, Mostgaard G, Oxhoj H (1994). Evaluation of peak expiratory flow variability in an adolescent population sample. The Odense Schoolchild Study. Am J Respir Crit Care Med.

[18] Croese TJ (1988). Eosinophilic enteritis – a recent north Queensland experience. Aust N Z J Med.

[19] Egbagbe E, Pavord ID, Wilding P, Thompson-Coon J, Tattersfield AE (1997). Adenosine monophosphate and histamine induced bronchoconstriction: repeatability and protection by terbutaline. Thorax.

[20] Cockcroft DW, Killian DN, Mellon JJ, Hargreave FE (1977). Bronchial reactivity to inhaled histamine: a method and clinical survey. Clin Allergy.

[21] Croese J, Speare R (2006). Intestinal allergy expels hookworms: seeing is believing. Trends Parasitol.

[22] Calvert J, Burney P (2005). Effect of body mass on exercise-induced bronchospasm and atopy in African children. J Allergy Clin Immunol.

[23] Palmer LJ, Celedon JC, Weiss ST, Wang B, Fang Z, Xu X (2002). *Ascaris lumbricoides* infection is associated with increased risk of childhood asthma and atopy in rural China. Am J Respir Crit Care Med.

[24] Lynch NR, Palenque M, Hagel I, DiPrisco MC (1997). Clinical improvement of asthma after anthelminthic treatment in a tropical situation. Am J Respir Crit Care Med.

[25] Girod N, Brown A, Pritchard DI, Billett EE (2003). Successful vaccination of BALB/c mice against human hookworm (*Necator americanus*): the immunological phenotype of the protective response. Int J Parasitol.

[26] Maxwell C, Hussain R, Nutman TB (1987). The clinical and immunologic responses of normal human volunteers to low dose hookworm (*Necator americanus*) infection. Am J Trop Med Hyg.

[27] van Grunsven PM, van Schayck CP, Molema J, Akkermans RP, van Weel C (1999). Effect of inhaled corticosteroids on bronchial responsiveness in patients with “corticosteroid naive” mild asthma: a meta-analysis. Thorax.

[28] Britton J, Hanley SP, Garrett HV, Hadfield JW, Tattersfield AE (1988). Dose related effects of salbutamol and ipratropium bromide on airway calibre and reactivity in subjects with asthma. Thorax.

[29] Huang SL, Tsai PF, Yeh YF (2002). Negative association of Enterobius infestation with asthma and rhinitis in primary school children in Taipei. Clin Exp Allergy.

[30] Preston PJ (1970). The biology of the atopic response. J R Nav Med Serv.

[31] Turton JA (1976). Letter: IgE, parasites, and allergy. Lancet.

[32] Pritchard DI, Moqbel R (1992). Parasites and allergic disease: a review of the field and experimental evidence for a ‘cause-and-effect’ relationship. Allergy and immunity to helminthes.

[33] Turner KJ, Feddema L, Quinn EH (1979). Non-specific potentiation of IgE by parasitic infections in man. Int Arch Allergy Appl Immunol.

